# One-step genetic correction of hemoglobin E/beta-thalassemia patient-derived iPSCs by the CRISPR/Cas9 system

**DOI:** 10.1186/s13287-018-0779-3

**Published:** 2018-02-26

**Authors:** Methichit Wattanapanitch, Nattaya Damkham, Ponthip Potirat, Kongtana Trakarnsanga, Montira Janan, Yaowalak U-pratya, Pakpoom Kheolamai, Nuttha Klincumhom, Surapol Issaragrisil

**Affiliations:** 1 Siriraj Center of Excellence for Stem Cell Research, Faculty of Medicine Siriraj Hospital, Mahidol University, Bangkok, Thailand; 2 Department of Research and Development, Faculty of Medicine Siriraj Hospital, Mahidol University, Bangkok, Thailand; 3 Department of Biochemistry, Faculty of Medicine Siriraj Hospital, Mahidol University, Bangkok, Thailand; 4 Division of Hematology, Department of Medicine, Faculty of Medicine Siriraj Hospital, Mahidol University, Bangkok, Thailand; 5 Division of Cell Biology, Department of Pre-clinical Sciences, Faculty of Medicine, Thammasat University, Pathumthani, Thailand; 6 Department of Anatomy, Faculty of Dentistry, Chulalongkorn University, Bangkok, Thailand

**Keywords:** Induced pluripotent stem cells, Thalassemia, Hematopoietic differentiation, Genetic correction, CRISPR/Cas9

## Abstract

**Background:**

Thalassemia is the most common genetic disease worldwide; those with severe disease require lifelong blood transfusion and iron chelation therapy. The definitive cure for thalassemia is allogeneic hematopoietic stem cell transplantation, which is limited due to lack of HLA-matched donors and the risk of post-transplant complications. Induced pluripotent stem cell (iPSC) technology offers prospects for autologous cell-based therapy which could avoid the immunological problems. We now report genetic correction of the beta hemoglobin (*HBB*) gene in iPSCs derived from a patient with a double heterozygote for hemoglobin E and β-thalassemia (HbE/β-thalassemia), the most common thalassemia syndrome in Thailand and Southeast Asia.

**Methods:**

We used the CRISPR/Cas9 system to target the hemoglobin E mutation from one allele of the *HBB* gene by homology-directed repair with a single-stranded DNA oligonucleotide template. DNA sequences of the corrected iPSCs were validated by Sanger sequencing. The corrected clones were differentiated into hematopoietic progenitor and erythroid cells to confirm their multilineage differentiation potential and hemoglobin expression.

**Results:**

The hemoglobin E mutation of HbE/β-thalassemia iPSCs was seamlessly corrected by the CRISPR/Cas9 system. The corrected clones were differentiated into hematopoietic progenitor cells under feeder-free and OP9 coculture systems. These progenitor cells were further expanded in erythroid liquid culture system and developed into erythroid cells that expressed mature *HBB* gene and HBB protein.

**Conclusions:**

Our study provides a strategy to correct hemoglobin E mutation in one step and these corrected iPSCs can be differentiated into hematopoietic stem cells to be used for autologous transplantation in patients with HbE/β-thalassemia in the future.

**Electronic supplementary material:**

The online version of this article (10.1186/s13287-018-0779-3) contains supplementary material, which is available to authorized users.

## Background

HbE/β-thalassemia, a double heterozygosity of hemoglobin E (HbE) and β-thalassemia, is the most common thalassemic syndrome found in adults in Southeast Asia. The clinical manifestations are heterogeneous; at one end the mutation may be very mild, whereas at the other end it is very severe similar to homozygous β-thalassemia or thalassemia major. In HbE/β-thalassemia, one allele (β^0^) produces no β-globin chain and the other allele (β^E^) produces a HbE globin chain resulting from nucleotide substitution at codon 26 (GAG → AAG, glutamic acid to lysine) [1]. Previous reports have shown the success of genetic correction of a β-globin gene in β-thalassemia-specific induced pluripotent stem cells (iPSCs) using a lentiviral vector [2, 3] or homologous recombination [4]. However, the lentiviral gene therapy results in random integration of the functional gene into the genome, leading to undesired mutations. It is therefore necessary to screen for the clones that integrate the transgene to genomic safe harbor sites. On the other hand, the classic homologous recombination is very inefficient, especially in human pluripotent stem cells [5]. The more efficient genome editing technologies using custom-engineered nucleases, zinc-finger nucleases (ZFNs) and transcription activator-like effector nucleases (TALENs), have been used to correct β-thalassemia iPSCs [6, 7].

The RNA-guided clustered regularly interspaced short palindromic repeat (CRISPR)/Cas9 system has been used recently to correct *HBB* mutation in iPSCs derived from β-thalassemia [8–11] and sickle cell disease patients [12]. However, these studies relied on a donor plasmid containing a wild-type *HBB* gene and an antibiotic selection cassette for enrichment, thereby requiring subsequent excision and clonal selection steps. To overcome these limitations, a single-stranded DNA oligonucleotide (ssODN) donor template can be used to provide seamless correction [13, 14]. In this study, we used the CRISPR/Cas9 system and the ssODN donor template to efficiently correct the HbE mutation in iPSCs derived from a patient with HbE/β-thalassemia, resulting in the corrected iPSCs, which is a β-thalassemia heterozygote. The corrected iPSCs are capable of differentiating into hematopoietic stem cells, which can be used for autologous transplantation to the patient in the future. In addition, our study further demonstrates that these cells can differentiate in vitro to reticulocytes, which can be developed for therapeutic use.

## Methods

### Sample collection and generation of induced pluripotent stem cells

The study was approved by the Siriraj Institutional Review Board (no. Si248/2011), in accordance with the Helsinki Declaration of 1975. All patients were provided with an explanation and with a participant information sheet and signed the informed consent. Skin biopsies were collected from HbE/β-thalassemia patients for further mutation analysis and isolation of fibroblasts. Briefly, the skin specimens were washed with sterile phosphate buffered saline (PBS) containing 25 U/ml penicillin, 25 μg/ml streptomycin, chopped into small pieces of 1 mm^3^ and transferred into a T-25 tissue culture flask containing DMEM supplemented with 10% fetal bovine serum (FBS) (Lonza, Switzerland), 2 mM GlutaMAX™ and 25 U/ml penicillin, 25 μg/ml streptomycin. Fibroblasts were subcultured once every 5 days or whenever they reached 80% confluency by incubation with 0.25% Trypsin for 2 min. Generation and characterization of Eβ-iPSCs from a HbE/β-thalassemic patient’s HDFs were performed as described previously [15]. iPSCs were maintained in mTeSR™1 medium (StemCell Technologies, Canada) on Matrigel™-coated (BD Bioscience, USA) plates and subcultured using 1 mg/ml Dispase (StemCell Technologies) according to the manufacturer’s instructions.

### Gene expression analysis

Total RNA was obtained using TRIzol® reagent (Invitrogen). cDNA was prepared using 2 μg of RNA and reverse-transcribed using the SuperScript III First-Strand Synthesis System and Oligo (dT) primers (Invitrogen). PCR analysis of pluripotent genes was performed on a T100™ Thermal Cycler (Bio-Rad, USA) using Platinum Taq DNA polymerase (Invitrogen). Primer sequences are presented in Additional file 1: Table S1. For quantitative RT-PCR (qRT-PCR) analysis, primers and probes were designed using the Universal Probe Library Assay Design Center (Roche Diagnostics). The qRT-PCR analysis was carried out on the CFX96™ Real-Time PCR detection system (Bio-Rad). Data were normalized with a housekeeping gene, *GAPDH*, and expressions were plotted against the undifferentiated normal iPSCs. Primer sequences are presented in Additional file 1: Table S2.

### Multiplex PCR analysis for hemoglobin E

Genomic DNA was isolated using the Gentra® Puregene® Cell Kit (Qiagen) according to the manufacturer’s instructions. HbE mutation was detected by multiplex PCR. The PCR reaction consisted of 1.5 U of DNA polymerase (Platinum Taq; Invitrogen), 1× PCR buffer, 1.5 mM MgCl_2_, 0.1 mM dNTPs, 0.4 μM HbE-Fc primer, 0.4 μM HbE-Rc primer, 0.5 μM HbE-Rn primer, 0.5 μM HbE-Fm primer and 2 μl of DNA sample in a total volume of 30 μl. The PCR was performed after an initial denaturation at 95 °C for 15 min followed by 30 cycles of denaturation (94 °C for 45 s), annealing (68 °C for 45 s) and extension (72 °C for 1 min), and a final extension step (72 °C for 7 min). Primer sequences are presented in Additional file 1: Table S3.

### Generation of CRISPR/Cas plasmid, single-guide RNA and single-stranded DNA oligonucleotide template

CRISPR/Cas plasmids pSpCas9(BB)-2A-GFP (PX458) and pSpCas9(BB)-2A-Puro (PX459) were obtained from Addgene (Cambridge, USA). Seven gRNAs targeting the HbE mutation were designed and their potential off-target sites were identified using the Crispr Design Tool (http://crispr.mit.edu/). Top and bottom strands of each gRNA were annealed, phosphorylated and cloned into the *Bbs*I site of the PX459 plasmid according to a protocol published previously [16]. The ssODN donor template for HbE correction was designed to have homology arms of 90 nucleotides on either side of the point mutation (a total of 181 nucleotides). The gRNA sequences and the ssODN template are presented in Additional file 1: Tables S4 and S5.

### Gene targeting of the HbE mutation in Eβ-iPSCs

Transfection of Eβ-iPSCs was performed using Amaxa 4D-nucleofector as described previously [17]. Briefly, 5 × 10^5^ Eβ-iPSCs were resuspended in nucleofection mixture containing 20 μl of P3 Primary Cell Solution (Lonza) and 2 μg of DNA. The mixture was transferred to Nucleocuvette strips and nucleofection was performed using the CB-150 program according to the manufacturer’s instructions. The PX459 plasmid was used for gRNA construction and the PX458 was used as a positive control. The transfected iPSCs were plated onto Matrigel™-coated 24-well plates and cultured in mTESR1 medium supplemented with 10 μM Y-27632. At 24 hours post nucleofection, the PX458 transfected cells were subjected to flow cytometry analysis and the percentage of GFP^+^ cells was analyzed by FACSCalibur™ (BD Biosciences). To determine the gRNA efficiency, the genomic DNA of PX459-gRNA transfected cells was extracted after being cultured for 5 days. The PCR product of 306 bp around the target site was amplified and digested with T7 endonuclease I (T7EI) enzyme (New England Biolabs) according to the manufacturer’s instructions.

To target the HbE mutation in Eβ-iPSCs, 200 pmol of the ssODN template was cotransfected with 2 μg of the PX459-gRNA with the highest specificity to the HbE site, as determined by T7EI assay. At 3 days post nucleofection, we performed a clonal isolation by limiting dilution. Briefly, transfected iPSCs were pretreated with SMC4 (Corning) prior to dissociation with accutase (Merck) and plated into 96-well plates at a density of 20 cells/96-well plate in mTESR1 medium supplemented with SMC4 for 8 days. Single colonies were picked and screened for the HbE mutation. The DNA sequence of HbE negative clones was further confirmed by direct sequencing. For off-target analysis, five potential off-target sites were amplified by PCR. In brief, genomic DNA was extracted using QuickExtract DNA Extraction Solution (Epicentric) according to the manufacturer’s instructions. The PCR reaction consisted of 0.2 U of Q5 High-Fidelity DNA polymerase (New England Biolabs), 1× Q5 PCR buffer with MgCl_2_, 0.2 mM dNTPs, 0.5 μM of each primer and 2 μl of DNA sample in a total volume of 50 μl. The PCR was performed with an initial denaturation at 98 °C for 30 s followed by 35 cycles of denaturation (98 °C for 10 s), annealing (68 °C for 30 s) and extension (72 °C for 30 s), followed by a final extension step (72 °C for 2 min). Primer sequences are presented in Additional file 1: Table S6. DNA sequences were validated by Sanger sequencing.

### Hematopoietic differentiation of iPSCs to myeloid and erythroid lineages

Hematopoietic differentiation was performed according to the previous study [18]. Briefly, iPSCs were subcultured on Matrigel™-coated six-well plates at 20–30% confluency in mTESR1 medium. After 24 hours of culture (day 0), the medium was replaced with differentiation medium: day 0–1 medium, RPMI (Gibco) supplemented with 5 ng/ml of human bone morphogenetic protein 4 (hBMP4) (Peprotech), 50 ng/ml of human vascular endothelial growth factor (hVEGF) (Peprotech), 25 ng/ml of hWnt3a (R&D) and 5% Knockout serum replacement (KOSR) (Gibco); day 2 medium, RPMI supplemented with 5 ng/ml of hBMP4, 50 ng/ml of hVEGF, 20 ng/ml basic fibroblast growth factor (bFGF) (Peprotech) and 5% KOSR; day 3 medium, StemPro-34 (Invitrogen), 5 ng/ml of hBMP4, 50 ng/ml of hVEGF and 20 ng/ml bFGF; day 4–5 medium, StemPro-34, 15 ng/ml of hVEGF and 5 ng/ml bFGF; day 6 medium, 74% Iscove modified Dulbecco medium (IMDM) (Gibco), 24% Ham's F12 (Gibco), 1% B27 supplement (Gibco), 0.5% N2 supplement (Gibco), 0.5% bovine serum albumin (BSA), 50 ng/ml of hVEGF, 100 ng/ml of bFGF, 100 ng/ml of human stem cell factor (SCF) (R&D) and 25 ng/ml of hFlt3 ligand (R&D); and day 7 medium, 74% IMDM, 24% Ham's F12, 1% B27 supplement, 0.5% N2 supplement, 0.5% BSA, 50 ng/ml of hVEGF, 100 ng/ml of bFGF, 100 ng/ml of hSCF, 25 ng/ml of hFlt3 ligand, 50 ng/ml of human thrombopoietin (TPO) (R&D), 10 ng/ml of IL-6 (Peprotech), 0.5 U/ml of hEPO (Eprex) and 0.2 μM of 6-formylindolo[3,2-b]carbazole (FICZ) (Abcam). After day 7, 0.5 ml of day 7 medium was added to the culture daily without removing the medium. All basal media mixes included 2 mM of GlutaMAX™, 0.4 mM of monothioglycerol (MTG) (Sigma-Aldrich), 100 μg/ml of Primocin (Invivogen) and 50 μg/ml of ascorbic acid (Sigma-Aldrich). Differentiated cells on days 10–14 were collected for gene expression and flow cytometry analysis. Adherent cells were dissociated with accutase at 37 °C for 15 min, washed twice with PBS/EDTA + 2% FBS and stained with antibodies specific to hematopoietic markers (CD34-PE, CD43-FITC, CD235a-PE and CD71-FITC; all from BioLegend) at room temperature for 15 min. The stained cells were washed twice with PBS/EDTA+ 2% FBS before being fixed with 300 μl of 1% paraformaldehyde and analyzed by FACSCalibur and CELLQuest software (BD Biosciences, USA). The floating cells on day 12 were also collected for colony-forming unit (CFU) assay by culturing in the methylcellulose-based medium, MethoCult™ H4435 Enriched (StemCell Technologies). The CFU number was analyzed after 14 days of culture.

Alternatively, iPSCs were also differentiated on OP9 mouse stromal cells. Briefly, small clumps of iPSCs were seeded onto overgrown OP9 cells in differentiation medium containing α-MEM (Invitrogen), 10% defined FBS (Hyclone), 100 mM MTG and 25 U/ml penicillin, 25 μg/ml streptomycin for 5 days. A half medium change was performed on day 3. iPSCs were harvested on day 6 using type IV collagenase (Invitrogen) for 20 min, followed by 0.25% trypsin for 15 min. CD34^+^ cells were isolated from differentiated cells using EasySep (StemCell Technologies) according to the manufacturer’s instructions. To promote erythroid maturation, the purified CD34^+^ cells were cultured in a three-stage culture system according to Griffiths et al. [19]. Briefly, cells were seeded in stage 1 medium containing basic medium (IMDM (Biochrom), 3% AB serum, 2% defined FBS, 10 μg/ml insulin, 3 U/ml heparin, 3 U/ml EPO, 200 μg/ml transferrin and 100 U/ml penicillin/streptomycin) supplemented with 10 ng/ml SCF and 1 ng/ml IL-3 for 8 days, stage 2 medium containing basic medium supplemented with 10 ng/ml SCF for 3 days and stage 3 medium containing basic medium supplemented with extra transferrin to a final concentration of 500 μg/ml for 13 days. Morphological analysis was performed by Wright’s staining at the indicated time points. The differentiated cells (1.5 × 10^4^–3 × 10^4^) were spun onto glass slides at 1000 rpm for 5 min using a Cytospin™ 4 Cytocentrifuge (Thermo Scientific) and stained with Wright-Giemsa (Merck). Images were taken with a light microscope (Carl Zeiss, Axio Star Plus).

### Western blotting analysis

Differentiated iPSCs at day 24 of erythroid liquid culture were harvested, washed with PBS and lysed in lysis buffer (Cell Signaling) for 1 hour on ice. The protein concentration was measured by the Pierce™ BCA Protein Assay Kit (Thermo Scientific). Samples were loaded onto 18% SDS-PAGE and transferred to a PVDF membrane. The membrane was blocked with 10% skimmed milk in Tris-buffered saline (TBS) with 0.1% Tween-20 (TBS-T) for 1 hour at room temperature and probed with the primary antibodies, anti-hemoglobin alpha and anti-hemoglobin beta (Santa Cruz) overnight at 4 °C. Subsequently, the membrane was incubated with the HRP-conjugated secondary antibody for 1 hour at room temperature. After washing with TBS-T, the membrane was incubated with ECL substrate (Thermo Scientific) and the results were visualized by an ImageQuant LAS 4010 Biomolecular imager (GE Healthcare).

## Results

### Generation and characterization of HbE/β-thalassemia patient-specific iPSCs

**Fig. 1:**
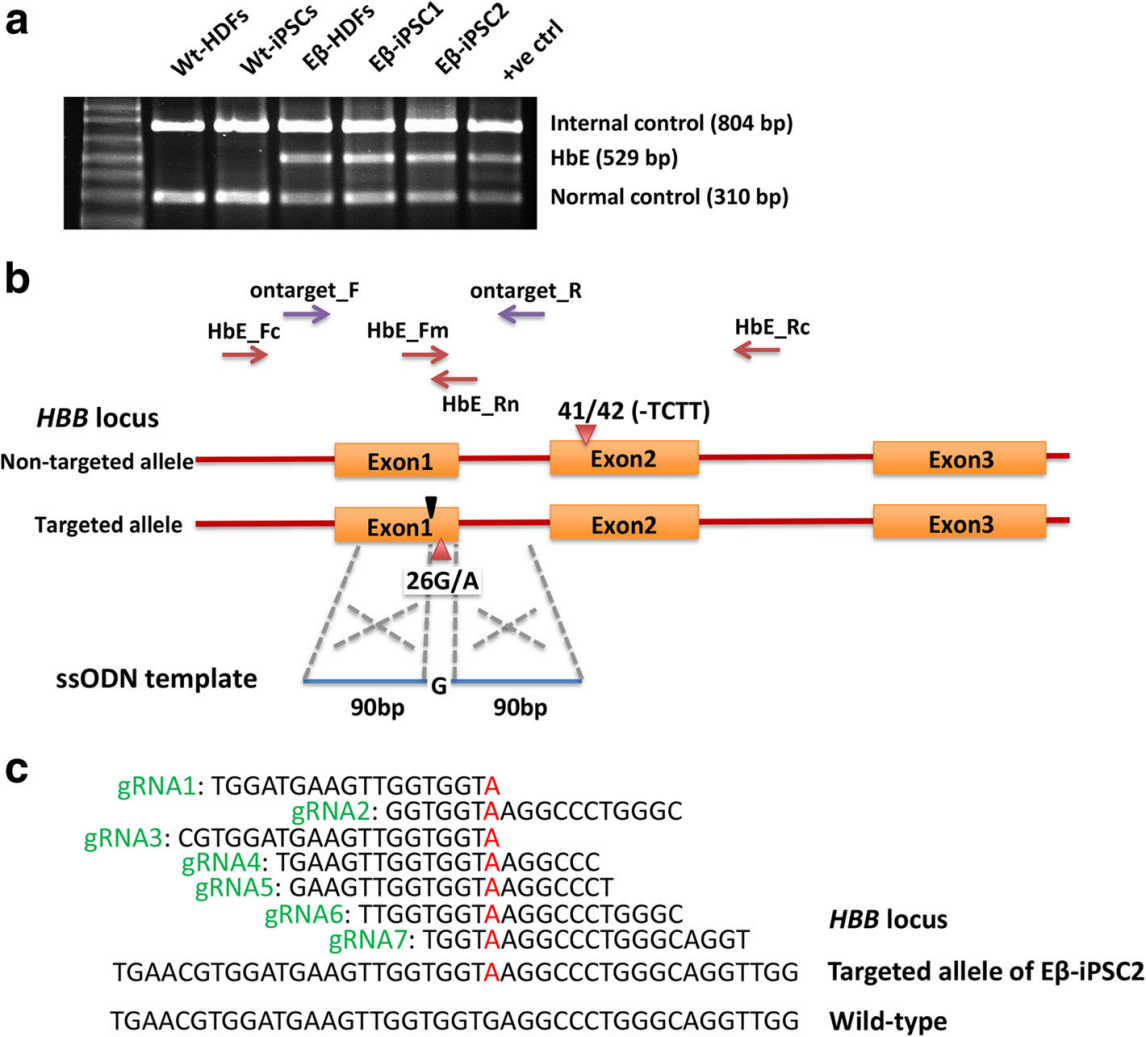
Strategy to target HbE mutation in Eβ-iPSCs using CRISPR/Cas9 to induce a double-stranded break at the *HBB* locus and a single-stranded oligo donor (ssODN) template to repair the mutation. **a** Multiplex PCR analysis for HbE mutation. wt-HDFs and wt-iPSCs indicate wild-type human dermal fibroblasts and iPSCs from a healthy individual. Eβ-HDFs, Eβ-iPSC1 and Eβ-iPSC2 are human dermal fibroblasts and two iPSC lines derived from a patient with HbE/β**-**thalassemia. **b** Schematic of targeted region of the *HBB* locus of the patient with HbE/β**-**thalassemia. The patient has a 4-bp deletion (–TCTT) in one allele and a point mutation at codon 26 (G → A) resulting in a structural variant hemoglobin E (HbE) in the other. Seamless and efficient correction of HbE is achieved using a gRNA targeting the point mutation (A) and the ssODN template carrying the correct nucleotide (G) with the left and right homology arms of 90 bp. Orange boxes indicate exons; red lines indicate introns. Red arrowheads show mutation sites; black arrowhead shows cleavage site of Cas9 by gRNA1; purple and red arrows indicate primer pairs for T7E1 assay and multiplex PCR for HbE detection, respectively. **c** Sequence of seven gRNAs designed to target the HbE mutation. Red “A” indicates point mutation in the Eβ-iPSC2 cells and gRNAs at *HBB* locus. HBB beta hemoglobin

We obtained human dermal fibroblasts (HDFs) from a patient with HbE/β**-**thalassemia who had a 4-bp deletion (–TCTT) at codon 41/42 in one allele, and a point mutation (G → A) at codon 26 resulting in abnormal hemoglobin E (HbE) production in the other, and generated iPSCs from these HbE/β**-**thalassemia patient’s HDFs. Several iPSC lines, designated Eβ-iPSCs, were obtained. Of these, two iPSC lines (Eβ-iPSC1 and Eβ-iPSC2) were expanded and characterized. To confirm the HbE mutation, multiplex PCR analysis was performed with the genomic DNA of the Eβ-iPSC1 and the Eβ-iPSC2 cells. Both Eβ-iPSCs had a product size of 529 bp, which represents the HbE point mutation, similar to that of fibroblasts derived from the patient’s skin (Eβ-HDFs) but not in the wild-type HDFs or iPSCs (Fig. 1a). Further characterization showed that both Eβ-iPSCs expressed pluripotent markers and were able to differentiate both in vitro and in vivo into cells/tissues of three embryonic germ layers. The Eβ-iPSCs exhibited a normal karyotype of 46, XY at passage 19 (Additional file 2: Figure S1). We then selected the Eβ-iPSC2 cells for further analyses.

### Correction of HbE mutation in Eβ-iPSC2 cells using the CRISPR/Cas9 system

**Fig. 2:**
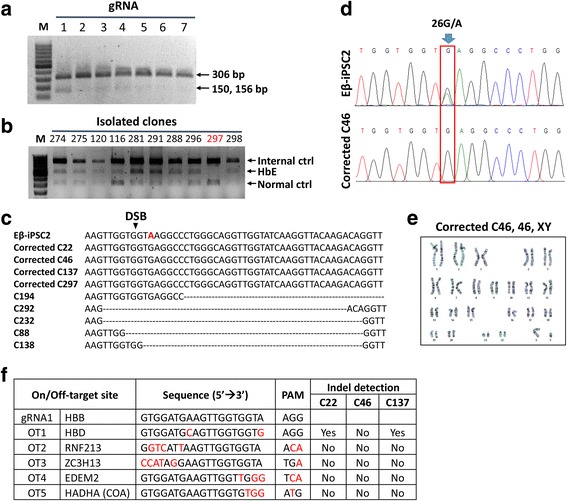
Genetic correction of HbE mutation of the *HBB* gene. **a** T7E1 assay of gRNA target sites. At 5 days post transfection, genomic DNA of PX459-gRNA transfected cells was extracted and the region spanning the gRNA target sites was PCR amplified using on-target primer pairs (arrows), giving PCR products of 306 bp (uncut) and 150 and 156 bp (cut). M indicates marker. **b** Multiplex PCR screening for HbE mutation of isolated clones after genetic correction by CRISPR/Cas9 and the ssODN template. HbE-negative clone (clone 297) indicated in red. **c** Representative DNA sequences of PCR products of the region spanning the gRNA target site in HbE-negative clones with HDR in the corrected clones and indels in others. DSB indicates the double-stranded break site generated by gRNA1. **d** Chromatogram of the parental Eβ-iPSC2 cells and the corrected C46 cells at the mutation site. Red box indicates HbE mutation (G → A) at codon 26. Note the overlapped peaks in the Eβ-iPSC2 cells, which occurred as a result of G in one allele (normal) and A in another allele (HbE). After genetic correction, both alleles contained the right nucleotide “G”. **e** Representative karyotype of the corrected C46 cells, which exhibited a normal karyotype (46, XY). **f** Potential off-target sites for gRNA1 as identified by BLAST search. Mismatch nucleotides are indicated in red. Eβ-iPSC2 iPSC lines derived from a patient with HbE/β**-**thalassemia, HbE hemoglobin E, HBD delta hemoglobin

To correct the mutation in the Eβ-iPSC2 cells, guide RNAs (gRNAs) were designed to target the HbE mutation at codon 26 where nucleotide substitution occurs (G → A) (Fig. 1b). We first designed seven 20-nucleotide (nt) or truncated (18-nt or 19-nt) gRNAs targeting regions close to the point mutation (Fig. 1c and Additional file 1: Table S4). Each gRNA was constructed into a PX459 plasmid, which contains the Cas9 nuclease and puromycin selection cassettes. We used PX458, which has the same backbone as PX459 except for GFP expression, instead of the puromycin selection cassette, to determine the transfection efficiency. At 24 hours post nucleofection, the PX458 transfected cells were harvested for flow cytometry analysis. We obtained approximately 30% transfection efficiency in the Eβ-iPSC2 cells (Additional file 3: Figure S2). We then used the same conditions for transfecting PX459-gRNA into the Eβ-iPSC2 cells. We harvested the genomic DNA of the PX459-gRNA transfected pool at 5 days post nucleofection and examined for gRNA-Cas9 cleavage efficiency and specificity using T7EI assay. Of these seven gRNAs, gRNA1, which targeted DSB at two nucleotides upstream of the HbE point mutation, gave the highest efficiency and was then selected for further experiments (Fig. 2a).

For HbE correction, we cotransfected PX459-gRNA1 and the ssODN template. At 3 days post nucleofection, the transfected pool was harvested and seeded at a density of 20 cells/well of a 96-well plate. Eight to 10 days after plating, one to five colonies were observed in each well. Single colonies were individually picked for multiplex PCR analysis for HbE mutation. From a total of 312 individual clones screened, we estimated that 93 clones were transfected (according to 30% transfection efficiency with the GFP plasmid control). We obtained 23 HbE-negative clones from PCR screening (7.4% DSB efficiency) (Fig. 2b). Of these, 14 clones showed indels (4.5% nonhomologous end joining (NHEJ)) and nine clones showed successful seamless correction of HbE mutation (2.9% homology-directed repair (HDR)) as confirmed by direct sequencing (Fig. 2c, d). We selected five clones (C22, C46, C134, C137 and C258) for karyotyping analysis. These corrected clones exhibited a normal karyotype (Fig. 2e and Additional file 4: Figure S3) and three clones (C22, C46 and C137) were randomly expanded for off-target analysis.

### Off-target analysis

We selected five potential off-target sites of gRNA1 including the *HBD* gene and other genes that have similar homology with the gRNA1-PAM sequence using NCBI BLAST. PCR amplification of these regions was performed and DNA sequences of the PCR products were validated by direct sequencing as compared to that of the Eβ-iPSC2 cells. We detected no off-target cleavage in the corrected C46 cells, whereas a point mutation in the *HBD* gene was observed in both of the corrected C22 and C137 cells (Fig. 2f).

### Generation of hematopoietic cells from the corrected iPSCs

**Fig. 3:**
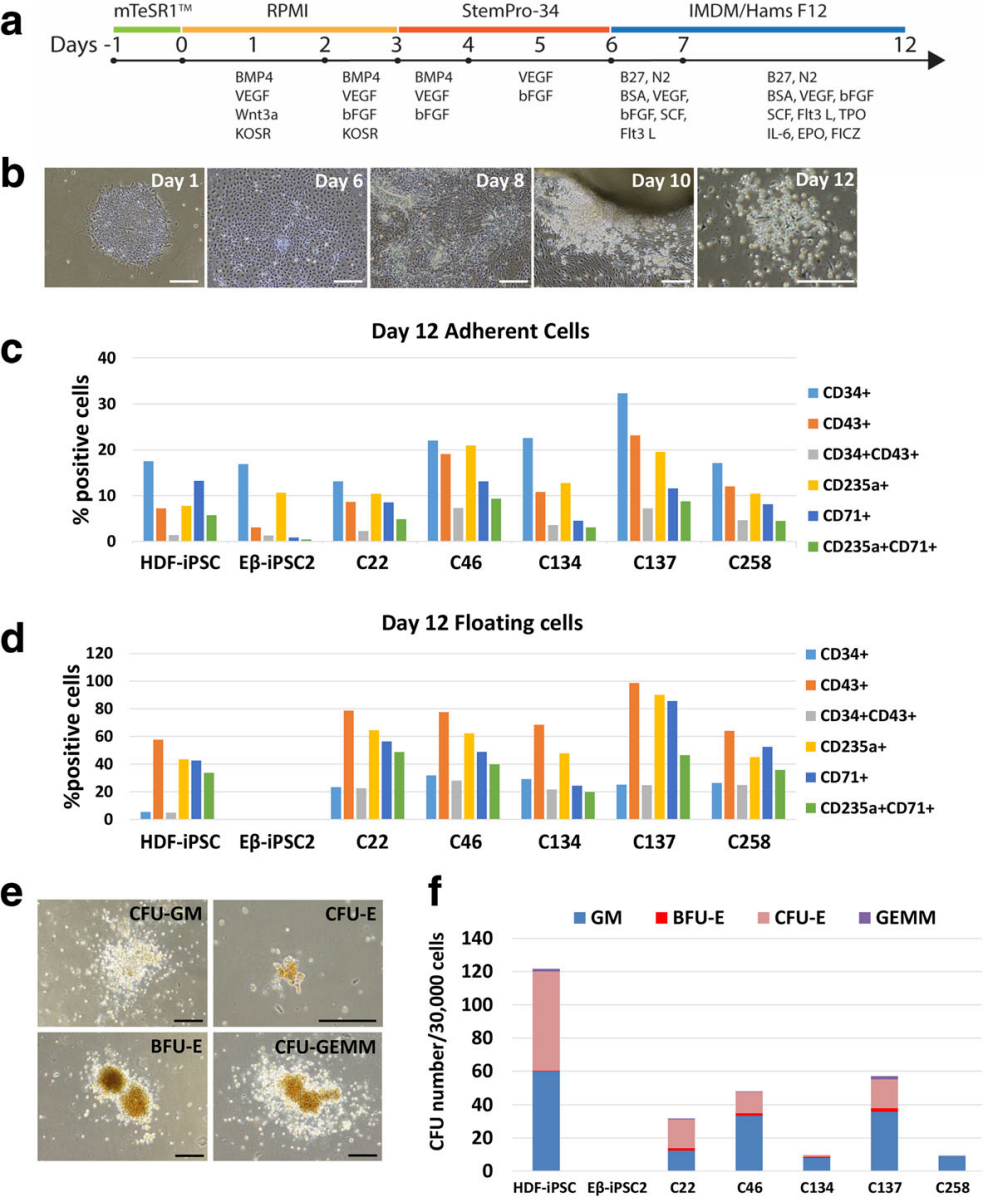
Hematopoietic differentiation of iPSCs using the feeder-free system. **a** Schematic of the feeder-free hematopoietic differentiation protocol used in this study. **b** Morphological changes of the corrected C46 cells during hematopoietic differentiation. **c, d** Numbers of CD34, CD43, CD235a and CD71-expressing cells in adherent and nonadherent systems at day 12 of differentiation. **e** Representative images of CFU from HDF-iPSCs. Differentiated cells at day 12 were harvested and seeded in MethoCult media. **f** Numbers of CFU colonies counted on day 14 of culture in MethoCult media. Data obtained from two independent experiments. Scale bars = 200 μm. IMDM Iscove modified Dulbecco medium, BMP-4 bone morphogenetic protein 4, VEGF vascular endothelial growth factor, KOSR knockout serum replacement, bFGF basic fibroblast growth factor, BSA bovine serum albumin, SCF stem cell factor, TPO thrombopoietin, IL interleukin, FICZ 6-formylindolo[3,2-b]carbazole, HDF human dermal fibroblasts, iPSC induced pluripotent stem cell, Eβ-iPSC2 iPSC lines derived from a patient with HbE/β**-**thalassemia, CFU-E colony-forming unit erythroid, EPO erythropoietin, BFU-E burst-forming unit erythroid, GM granulocyte, macrophage, GEMM granulocyte, erythrocyte, macrophage, megakaryocyte

To evaluate whether the genetic correction in Eβ-iPSCs could restore the *HBB* expression, hematopoietic differentiation of the wild-type iPSCs (HDF-iPSCs), the Eβ-iPSC2 cells and the corrected clones (C22, C46, C134, C137 and C258) was performed in a feeder-free condition (Fig. 3a) at the following stages of hematopoietic development: mesoderm progenitor, hematovascular specification, endothelial hematopoietic transition and hematopoietic progenitor cells. At days 5–6 of culture, the differentiated cells appeared to be a monolayer of endothelial-like cells, which later formed three-dimensional structures, observed from day 8 onward. The nonadherent cells started to emerge from both monolayer and three-dimensional structures on days 8–12 (Fig. 3b). During hematopoietic differentiation, the Eβ-iPSC2 cells showed impaired hematopoietic differentiation as indicated by a lower number of cells expressing hematopoietic progenitor and erythroid markers, CD43 and CD71, when compared to the HDF-iPSCs in the adherent cell population. In contrast to the HDF-iPSCs, which could give rise to the nonadherent cell population that highly expressed CD43, CD71 and CD235, the Eβ-iPSC2 cells produced a very low number of nonadherent cells, which were mainly nonviable. After genetic correction, all of the five corrected clones were able to differentiate into hematopoietic progenitor cells which expressed CD34 and CD43, and erythroid markers CD71 and CD235a at comparable levels to those of the HDF-iPSCs in both adherent and nonadherent cell population (Fig. 3c, d). We also examined the gene expression profile of these corrected cells during hematopoietic specification by quantitative real-time PCR. All corrected clones expressed *SOX17* and *RUNX1*, which play an important role in blood formation from hemogenic endothelium, and *GATA1* and *KLF1*, which are erythroid-specific markers (Additional file 5: Figure S4a). We harvested the differentiated floating cells from the HDF-iPSCs and the corrected clones on day 12 and seeded them onto methylcellulose plates. After 2 weeks, the HDF-iPSCs and three of the corrected clones (C22, C46 and C137) gave rise to all types of colonies, mainly CFU-E and CFU-GM, confirming the functional characteristic of hematopoietic progenitor cells. However, two of the corrected clones (C134 and C258) could only give rise to CFU-GM and a small number of CFU-E (Fig. 3e, f). In contrast to the corrected cells, the Eβ-iPSC2 cells could not produce any CFU colonies. The BFU-E obtained from the corrected clones expressed high levels of fetal gamma hemoglobin (*HBG*) and low levels of adult beta hemoglobin (*HBB*) transcripts, when examined by qRT-PCR (Additional file 5: Figure S4b).

**Fig. 4:**
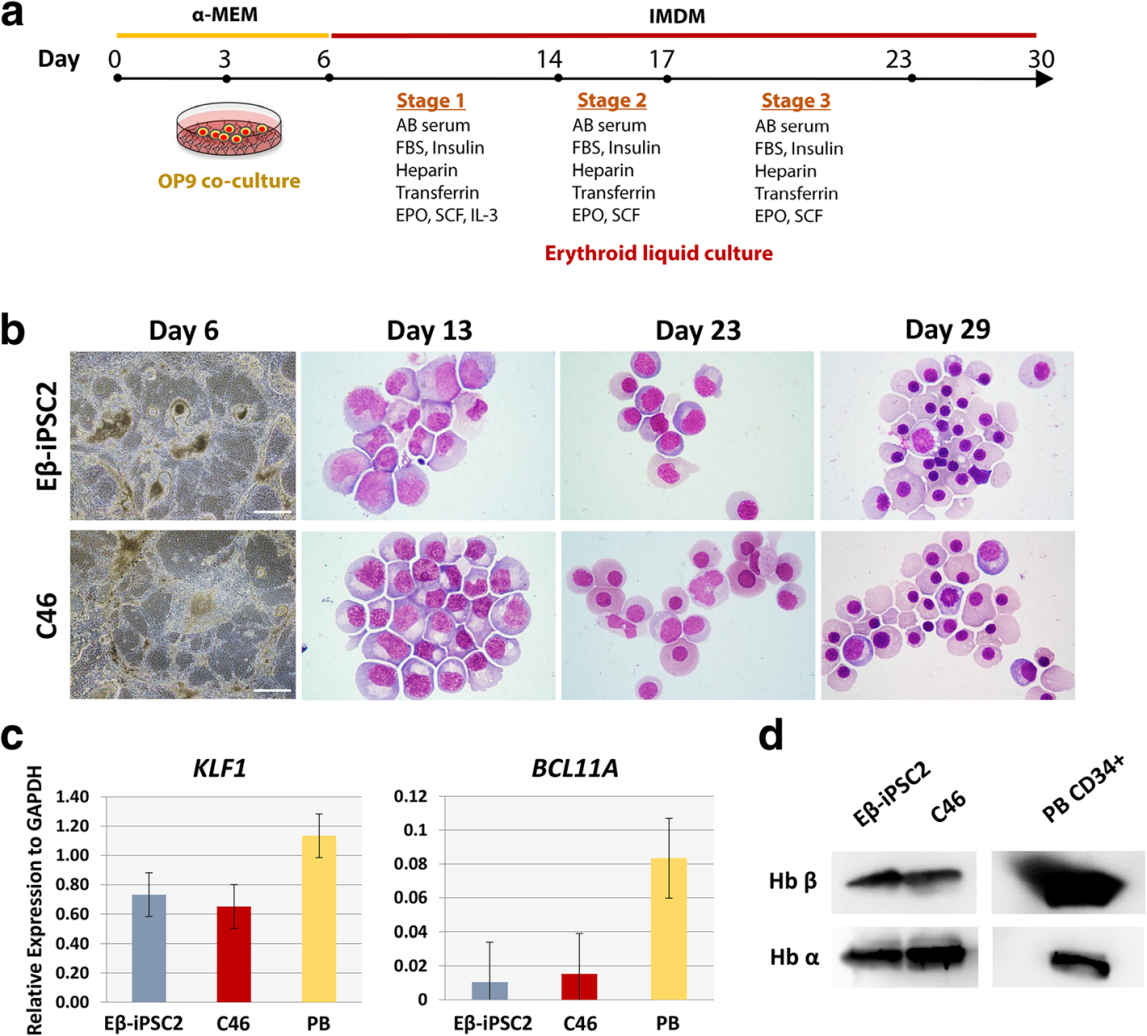
Hematopoietic differentiation of iPSCs using the OP9 coculture system and erythroid liquid culture. **a** Schematic of hematopoietic differentiation protocol used in this study. **b** Morphological changes of the Eβ-iPSC2 cells and the corrected C46 cells during hematopoietic differentiation on day 6 of OP9 coculture (scale bar = 500 μm), and Wright’s staining on days 13, 23 and 29 of differentiation. **c** Quantitative RT-PCR analysis of erythroid-associated transcription factors at day 19 of differentiation (equivalent to day 13 of erythroid liquid culture) of the Eβ-iPSC2 cells and the corrected C46 cells as compared to peripheral blood CD34^+^ cell-derived erythroblasts at day 13 (PB). Data presented as mean ± SD of triplicate samples from a representative experiment. **d** Western blot analysis of alpha and beta hemoglobin expression of the Eβ-iPSC2 cells and the corrected C46 cells at day 30 of differentiation as compared to peripheral blood CD34^+^ cell-derived erythroblasts at day 24 of erythroid liquid culture. MEM minimal essential medium, IMDM Iscove modified Dulbecco medium, FBS fetal bovine serum, SCF stem cell factor, IL interleukin, EPO erythropoietin, Eβ-iPSC2 iPSC lines derived from a patient with HbE/β**-**thalassemia, Hb hemoglobin

Since the Eβ-iPSC2 cells seemed to be refractory to the hematopoietic differentiation protocol under the feeder-free condition, we turned to the OP9 coculture system for hematopoietic differentiation followed by an erythroid liquid culture (Fig. 4a) [20]. The supportive OP9 stromal cells have been shown to efficiently induce hematopoietic differentiation [21]. We selected the corrected C46 cells, which differentiated well under the feeder-free condition and contained no off-target mutation, for comparison with the Eβ-iPSC2 cells. Small clumps of iPSCs were seeded onto overgrown OP9 cells and cultured for 6 days. In contrast to the feeder-free hematopoietic differentiation system, both the Eβ-iPSC2 cells and the corrected C46 cells were able to differentiate into sac-like structures (Fig. 4b). We isolated CD34^+^ cells from the differentiated cells on day 6 of the OP9 coculture system and further expanded erythroid cells using the three-stage culture system [19]. Upon erythroid culture, both the Eβ-iPSC2 cells and the corrected C46 cells gradually changed their morphology from that representing proerythroblasts/basophilic erythroblasts on day 13 of culture to that representing polychromatic/orthochromatic erythroblasts on day 23 and finally became orthochromatic erythroblasts/reticulocytes on day 29 of culture. Analysis of gene expression during the erythroid liquid culture demonstrated that both differentiated Eβ-iPSC2 and the corrected C46 cells at day 19 of differentiation (when the morphological stages are equivalent to those of day 13 erythroid cells derived from peripheral blood progenitors) expressed lower levels of erythroid-associated transcription factors *KLF1* and *BCL11A* as compared to the cultured erythroblasts from peripheral blood CD34^+^ cells (Fig. 4c). We harvested the differentiated cells at day 30 and analyzed hemoglobin protein expression. Both differentiated Eβ-iPSC2 and the corrected C46 cells expressed similar levels of beta hemoglobin and alpha hemoglobin proteins, indicating successful hematopoietic differentiation under the OP9 coculture system (Fig. 4d).

## Discussion

HbE is the hallmark of Southeast Asia. Double heterozygosity of HbE and β-thalassemia, namely HbE/β-thalassemia, can cause a severe clinical syndrome similar to homozygous β-thalassemia [22]. These severe patients manifest with anemia requiring red cell transfusion during the first year of life. The only curative therapy at present is hematopoietic stem cell transplantation. However, unavailability of HLA-matched donors and risk of transplant-related morbidity, mortality as well as immunologic complications, especially graft failure and graft versus host disease (GVHD), have limited the use of allogeneic transplantation [23]. Recently, in vitro genetic correction of hematopoietic stem cells (HSCs) has been successfully reported; however, culturing and maintaining HSCs remain difficult [24].

Advances in iPSC technology offer promise for autologous cell-based therapy as it provides a stem cell source, which can be continuously expanded in vitro and is amendable to genetic manipulation before differentiation into functional HSCs. We performed an efficient one-step seamless genetic correction of iPSCs from a HbE/β-thalassemic patient using the CRISPR/Cas9 system and the ssODN template. The genetic correction of the HbE mutation in one allele is easier than correcting the β-globin gene mutation in the other allele in which the mutations are heterogeneous [25]. Our approach does not require an antibiotic selection cassette that may interfere with expression of the corrected gene [6, 9, 12].

We designed gRNAs to recognize only HbE mutation and used the ssODN template possessing the correct nucleotide at the center of the oligo, which provides the highest targeting efficiency especially when the mutation site is less than 10 bp away from the cutting sites [26]. The ssODN template is easy to design and synthesize. It is footprint-free after genetic correction and has a high targeting efficiency. Our results show that transfection of PX459-gRNA1 with ssODN template resulted in a gene disruption frequency (DSB) of 7.4% and homologous gene targeting frequency (HDR) of 2.9%. Our transfection efficiency varied among iPSC lines, ranging from 30 to 60%. The number of screened clones could be further reduced if the transfection efficiency increased. To improve HDR efficiency, small molecule inhibitors may be used to suppress the nonhomologous end joining (NHEJ) pathway, thus facilitating the downstream screening process [27, 28].

Off-target mutagenesis is a major safety concern since genetic modifications are permanent and will have devastating consequences if the mutations are to be at important sites [29]. We identified five potential off-target sites of gRNA1 including the *HBD* gene, which has similar homology to *HBB* gene, and confirmed them by Sanger sequencing. Of the three corrected clones screened, one had no indel mutation whereas two harbored a point mutation. Interestingly, the presence of the point mutation in the delta hemoglobin (*HBD*) gene did not affect the hematopoietic differentiation potential when using the feeder-free system. A previous report on the genetic correction of a beta hemoglobin gene to treat sickle cell disease in human hematopoietic stem/progenitor cells using ZFNs showed off-target cleavage in the highly homologous, but functionally dispensable, *HBD* gene [30]. Measures to improve targeting specificity of Cas9 include the use of truncated gRNA with shorter regions of target complementarity [31], or modifying the Cas9 components so that two gRNA/Cas9 complexes are required to cleave DNA. The latter can be achieved via nickases that induce single-strand breaks [32, 33] or single-guide RNA (sgRNA)-guided catalytically inactive Cas9 (dCas9) fused to the *Fok*I nuclease [34–36]. Recently, an alternative CRISPR system to Cas9 nuclease called Cpf1 nuclease was used for genome editing with simpler crRNA synthesis and more effective delivery and targeting results [37]. Nevertheless, whole genome sequencing is still required before using these corrected clones in clinical applications.

Future therapeutic use of *ex vivo* genetically corrected HSCs will require efficient conversion of these stem cells to differentiated hematopoietic precursors and safety testing. We used two in vitro differentiation schemes to assess the potential of our cells for conversion to hematopoietic cells making normal hemoglobin products. We first induced hematopoietic differentiation using a chemically defined, serum and feeder cell-free protocol based on aryl hydrocarbon receptor (AhR) activation [18]. This protocol has been shown to facilitate an expansion of both erythroid and megakaryocyte progenitor cells. We compared the number of hematopoietic progenitor and erythroid markers of the wild-type iPSCs (HDF-iPSCs), the Eβ-iPSC2 cells and the five corrected clones. The number of adherent and nonadherent cells expressing those markers was comparable in the wild-type iPSCs and the five corrected clones; however, we noticed varying numbers of multilineage CFUs among these cell types due to clonal variability. In contrast, the Eβ-iPSC2 cells could differentiate to some extent, as demonstrated by the presence of CD34^+^ and CD71^+^ cells in adherent cells, although these cells could not give rise to CFUs. Hematopoietic differentiation based on the OP9 coculture system seemed to be more supportive for the Eβ-iPSC2 cells. Morphological changes of the Eβ-iPSC2 cells on the OP9 feeder layer were similar to those of the corrected C46 cells. Both cells produced significant numbers of erythroblasts with occasional enucleated mature erythrocytes using the three-stage erythroid liquid culture system. The erythroblasts from both the Eβ-iPSC2 cells and the corrected C46 cells expressed *KLF1* and *BCL11A* transcripts at lower levels compared to those of the peripheral blood-derived erythroblasts. Similarly, analysis of beta and alpha hemoglobin protein expressions showed that both the Eβ-iPSC2 cells and the corrected C46 cells expressed the proteins at similar levels; however, the beta hemoglobin levels were much lower than those of the peripheral blood-derived erythroblasts. A previous study demonstrated that the transcription factors KLF1 and BCL11A are required for the induction of beta hemoglobin levels [38]; therefore, low levels of *KLF1* and *BCL11A* transcripts in the corrected C46 cell-derived erythroid cells could result in lower beta hemoglobin expression. In addition, the number of erythroid cells derived from iPSCs is still limited; further optimization of the differentiation protocol to increase the number of erythroid cells should be performed to further facilitate an in vitro functional assay. In this study, the anti-human beta hemoglobin antibody could not distinguish the one amino acid difference from abnormal hemoglobin E in the Eβ-iPSC2 cells. Therefore, validation with other techniques such as HPLC should be performed to confirm the result at protein levels. In addition, further development of long-term repopulating hematopoietic stem/progenitor cells is required for successful engraftment in immunodeficient mouse models, where the clinical efficacy of corrected human hematopoietic stem cells can be more properly assessed.

## Conclusion

Our study provides a successful strategy to correct HbE mutation in one step and could be employed as a universal approach in the future correction of the *HBB* gene in iPSCs derived from other HbE/β^0^-thalassemia or β^+^**-**thalassemia patients. The results indicate that genetic correction of HbE mutation in one allele is sufficient to restore HBB protein expression upon hematopoietic differentiation by the OP9 coculture system followed by an erythroid liquid culture. Similarly, previous studies demonstrated that genetic correction of mutation in one allele—to a heterozygous state—in iPSCs derived from homozygous β-thalassemia or sickle cell disease was achieved using CRISPR/Cas9 or TALENs [6, 8, 9, 12]. Occasionally, homozygous corrections of homozygous β-thalassemia iPSCs were observed [10, 13]. The knowledge and protocols obtained from this study will facilitate and be applicable to genetic correction of patient-specific iPSCs with other genetic disorders.

## Additional files

Additional file 1: Tables S1–S6. Presenting primer and oligo sequences. (DOCX 20 kb)

Additional file 2: Figure S1. Showing characterization of iPSCs derived from skin fibroblasts of a patient with hemoglobin E/beta-thalassemia. (**a**) Pluripotent gene expression of wild-type human dermal fibroblasts (wt-HDFs), parental human dermal fibroblasts (Eβ-HDFs) and Eβ-iPSCs compared with hESC line Chula2.hES, analyzed by RT-PCR. (**b**) Immunofluorescent staining shows expression of pluripotent markers NANOG, OCT4, SSEA-4, TRA-1-60 and TRA-1-81 in the Eβ-iPSC1 and Eβ-iPSC2 cells. Scale bars = 100 μm. (**c**) Immunofluorescent staining shows expression of lineage markers NESTIN (ectoderm), AFP (endoderm) and SMA (mesoderm) of differentiated embryoid bodies generated from the Eβ-iPSC1 and Eβ-iPSC2 cells. Scale bars: for NESTIN and SMA = 100 μm; for AFP = 50 μm. (**d**) Hematoxylin and eosin (H&E) staining of teratomas derived from the Eβ-iPSC2 cells at 8 weeks post implantation into nude mice. Teratomas contained tissues derived from three embryonic germ layers, sebaceous tissue (ectoderm), cartilage (mesoderm) and gut-like epithelium (endoderm). Scale bars = 100 μm. (**e**) Representative karyotypic analysis of the Eβ-iPSC2 cells at passage 19 shows normal karyotype (46, XY) (TIFF 9760 kb)

Additional file 3: Figure S2. Showing transfection efficiency of PX458 in the Eβ-iPSC2 cells. (**a**) Phase contrast and fluorescent images of the Eβ-iPSC2 cells 1 day post transfection with PX458. (**b**) Flow cytometry analysis of GFP-expressing cells in the untransfected cells (negative control) and the PX458 transfected cells (TIFF 4645 kb)

Additional file 4: Figure S3. Showing representative karyotypes of the corrected C22, C134, C137 and C258 cells, which exhibited normal karyotypes (46, XY) (TIFF 3596 kb)

Additional file 5: Figure S4. Showing gene expression profile of the differentiated cells. (**a**) qRT-PCR analysis of hematopoietic and erythroid-specific markers: *RUNX1*, *SOX17*, *GATA1* and *KLF1*. Data presented as relative expression to day 0 of each sample, *N* = 2. (**b**) qRT-PCR analysis of fetal (*HBG*) and adult (*HBB*) globin gene expressions of day 0 normal iPSCs (D0 iPSC) and BFU-E and CFU-E colonies of the corrected C22 and C46 cells on day 14 of culture in MethoCult media. Data presented as relative expression to *GAPDH*, *N* = 2 (TIFF 1576 kb)

## Abbreviations

AhR: Aryl hydrocarbon receptor
BMP4: Bone morphogenetic protein 4
BSA: Bovine serum albumin
CFU: Colony-forming unit
CRISPR/Cas9: Clustered regularly interspaced short palindromic repeat/Cas9
FBS: Fetal bovine serum
FICZ: 6-Formylindolo[3,2-b]carbazole
gRNA: Guide RNA
GVHD: Graft versus host disease
HBB: Beta hemoglobin
HDF: Human dermal fibroblasts
HDR: Homology-directed repair
HSC: Hematopoietic stem cell
HSCT: Hematopoietic stem cell transplantation
IMDM: Iscove modified Dulbecco medium
iPSC: Induced pluripotent stem cell
KOSR: Knockout serum replacement
MTG: Monothioglycerol
NHEJ: Nonhomologous end joining
PBS: Phosphate buffered saline
SCF: Stem cell factor
ssODN: Single-stranded DNA oligonucleotide
TALEN: Transcription activator-like effector nuclease
TBS: Tris-buffered saline
TBST: Tris-buffered saline with 0.1% Tween-20
TPO: Thrombopoietin
VEGF: Vascular endothelial growth factor
ZFN: Zinc-finger nuclease

## References

[1] Olivieri NF, Pakbaz Z, Vichinsky E (2011). Hb E/beta-thalassaemia: a common & clinically diverse disorder. Indian J Med Res.

[2] Papapetrou EP, Lee G, Malani N, Setty M, Riviere I, Tirunagari LMS (2011). Genomic safe harbors permit high β-globin transgene expression in thalassemia induced pluripotent stem cells. Nat Biotechnol.

[3] Tubsuwan A, Abed S, Deichmann A, Kardel MD, Bartholoma C, Cheung A (2013). Parallel assessment of globin lentiviral transfer in induced pluripotent stem cells and adult hematopoietic stem cells derived from the same transplanted beta-thalassemia patient. Stem Cells.

[4] Wang Y, Zheng CG, Jiang Y, Zhang J, Chen J, Yao C (2012). Genetic correction of β-thalassemia patient-specific iPS cells and its use in improving hemoglobin production in irradiated SCID mice. Cell Res.

[5] Ding Q, Regan SN, Xia Y, Oostrom LA, Cowan CA, Musunuru K (2013). Enhanced efficiency of human pluripotent stem cell genome editing through replacing TALENs with CRISPRs. Cell Stem Cell.

[6] Ma N, Liao B, Zhang H, Wang L, Shan Y, Xue Y (2013). Transcription activator-like effector nuclease (TALEN)-mediated gene correction in integration-free beta-thalassemia induced pluripotent stem cells. J Biol Chem.

[7] Ma N, Shan Y, Liao B, Kong G, Wang C, Huang K (2015). Factor-induced reprogramming and zinc finger nuclease-aided gene targeting cause different genome instability in β-thalassemia induced pluripotent stem cells (iPSCs). J Biol Chem.

[8] Xie F, Ye L, Chang JC, Beyer AI, Wang J, Muench MO (2014). Seamless gene correction of beta-thalassemia mutations in patient-specific iPSCs using CRISPR/Cas9 and piggyBac. Genome Res.

[9] Song B, Fan Y, He W, Zhu D, Niu X, Wang D (2015). Improved hematopoietic differentiation efficiency of gene-corrected beta-thalassemia induced pluripotent stem cells by CRISPR/Cas9 system. Stem Cells Dev.

[10] Yang Y, Zhang X, Yi L, Hou Z, Chen J, Kou X (2016). Naïve induced pluripotent stem cells generated from β-thalassemia fibroblasts allow efficient gene correction with CRISPR/Cas9. Stem Cells Transl Med.

[11] Xu P, Tong Y, Liu X-Z, Wang T-T, Cheng L, Wang B-Y (2015). Both TALENs and CRISPR/Cas9 directly target the HBB IVS2–654 (C &gt; T) mutation in β-thalassemia-derived iPSCs. Sci Rep.

[12] Huang X, Wang Y, Yan W, Smith C, Ye Z, Wang J (2015). Production of gene-corrected adult beta globin protein in human erythrocytes differentiated from patient iPSCs after genome editing of the sickle point mutation. Stem Cells.

[13] Niu X, He W, Song B, Ou Z, Fan D, Chen Y (2016). Combining single-strand oligodeoxynucleotides and CRISPR/Cas9 to correct gene mutations in beta-thalassemia-induced pluripotent stem cells. J Biol Chem.

[14] Ou Z, Niu X, He W, Chen Y, Song B, Xian Y (2016). The combination of CRISPR/Cas9 and iPSC technologies in the gene therapy of Human β-thalassemia in mice. Sci Rep.

[15] Wattanapanitch M, Klincumhom N, Potirat P, Amornpisutt R, Lorthongpanich C, U-pratya Y (2014). Dual small-molecule targeting of SMAD signaling stimulates human induced pluripotent stem cells toward neural lineages. PLoS One.

[16] Ran FA, Hsu PD, Wright J, Agarwala V, Scott DA, Zhang F (2013). Genome engineering using the CRISPR-Cas9 system. Nat Protoc.

[17] Byrne SM, Mali P, Church GM (2014). Genome editing in human stem cells. Methods Enzymol.

[18] Smith BW, Rozelle SS, Leung A, Ubellacker J, Parks A, Nah SK (2013). The aryl hydrocarbon receptor directs hematopoietic progenitor cell expansion and differentiation. Blood.

[19] Griffiths RE, Kupzig S, Cogan N, Mankelow TJ, Betin VM, Trakarnsanga K (2012). Maturing reticulocytes internalize plasma membrane in glycophorin A-containing vesicles that fuse with autophagosomes before exocytosis. Blood.

[20] Trakarnsanga K, Wilson MC, Griffiths RE, Toye AM, Carpenter L, Heesom KJ (2014). Qualitative and quantitative comparison of the proteome of erythroid cells differentiated from human iPSCs and adult erythroid cells by multiplex TMT labelling and nanoLC-MS/MS. PLoS One.

[21] Slukvin II (2013). Hematopoietic specification from human pluripotent stem cells: current advances and challenges toward de novo generation of hematopoietic stem cells. Blood.

[22] Vichinsky E, Hemoglobin E (2007). Syndromes. ASH Education Program Book.

[23] Angelucci E, Matthes-Martin S, Baronciani D, Bernaudin F, Bonanomi S, Cappellini MD (2014). Hematopoietic stem cell transplantation in thalassemia major and sickle cell disease: indications and management recommendations from an international expert panel. Haematologica.

[24] Lengerke C, Daley GQ (2010). Autologous blood cell therapies from pluripotent stem cells. Blood Rev.

[25] Cao A, Galanello R (2010). Beta-thalassemia. Genet Med.

[26] Yang L, Guell M, Byrne S, Yang JL, De Los AA, Mali P (2013). Optimization of scarless human stem cell genome editing. Nucleic Acids Res.

[27] Robert F, Barbeau M, Ethier S, Dostie J, Pelletier J (2015). Pharmacological inhibition of DNA-PK stimulates Cas9-mediated genome editing. Genome Med.

[28] Chu VT, Weber T, Wefers B, Wurst W, Sander S, Rajewsky K (2015). Increasing the efficiency of homology-directed repair for CRISPR-Cas9-induced precise gene editing in mammalian cells. Nat Biotech..

[29] Cox DB, Platt RJ, Zhang F (2015). Therapeutic genome editing: prospects and challenges. Nat Med.

[30] Hoban MD, Cost GJ, Mendel MC, Romero Z, Kaufman ML, Joglekar AV (2015). Correction of the sickle cell disease mutation in human hematopoietic stem/progenitor cells. Blood.

[31] Fu Y, Sander JD, Reyon D, Cascio VM, Joung JK (2014). Improving CRISPR-Cas nuclease specificity using truncated guide RNAs. Nat Biotech..

[32] Ran FA, Hsu Patrick D, Lin C-Y, Gootenberg Jonathan S, Konermann S, Trevino AE (2013). Double nicking by RNA-guided CRISPR Cas9 for enhanced genome editing specificity. Cell.

[33] Mali P, Aach J, Stranges PB, Esvelt KM, Moosburner M, Kosuri S (2013). CAS9 transcriptional activators for target specificity screening and paired nickases for cooperative genome engineering. Nat Biotech..

[34] Guilinger JP, Thompson DB, Liu DR (2014). Fusion of catalytically inactive Cas9 to FokI nuclease improves the specificity of genome modification. Nat Biotech..

[35] Tsai SQ, Wyvekens N, Khayter C, Foden JA, Thapar V, Reyon D (2014). Dimeric CRISPR RNA-guided FokI nucleases for highly specific genome editing. Nat Biotech.

[36] Wyvekens N, Topkar VV, Khayter C, Joung JK, Tsai SQ (2015). Dimeric CRISPR RNA-guided FokI-dCas9 nucleases directed by truncated gRNAs for highly specific genome editing. Hum Gene Ther.

[37] Zetsche B, Gootenberg Jonathan S, Abudayyeh Omar O, Slaymaker Ian M, Makarova Kira S, Essletzbichler P (2015). Cpf1 is a single RNA-guided endonuclease of a class 2 CRISPR-Cas system. Cell.

[38] Trakarnsanga K, Wilson MC, Lau W, Singleton BK, Parsons SF, Sakuntanaga P (2014). Induction of adult levels of β-globin in human erythroid cells that intrinsically express embryonic or fetal globin by transduction with KLF1 and BCL11A-XL. Haematologica.

