# Supplementary material for: One-step genetic correction of hemoglobin E/beta-thalassemia patient-derived iPSCs by the CRISPR/Cas9 system
Source: Stem Cell Res Ther. 2018 Feb 26;9:46. doi: 10.1186/s13287-018-0779-3 (PMC5828150; doi:10.1186/s13287-018-0779-3)
Supplement: Supplementary file 1 — Presenting primer and oligo sequences. (DOCX 20 kb) [file 13287_2018_779_MOESM1_ESM.docx]

**Table S1. Primer sequences for RT-PCR analysis.**

| **Gene** | **Forward primer (5’🡪3’)** | **Reverse primer (5’🡪3’)** |
| --- | --- | --- |
| *NANOG* | CAGCCCCGATTCTTCCACCAGTCCC | CGGAAGATTCCCAGTCGGGTTCACC |
| *OCT4* | GACAGGGGGAGGGGAGGAGCTAGG | CTTCCCTCCAACCAGTTGCCCCAAAC |
| *SOX2* | GGGAAATGGGAGGGGTGCAAAAGAGG | TTGCGTGAGTGTGGATGGGATTGGTG |
| *GAPDH* | GTCAACGGATTTGGTCGTATTG | CATGGGTGGAATCATATTGGAA |

**Table S2. Real time PCR primers and probe numbers.**

| **Gene** |  | **Primer sequence (5’-3’)** | **Probe number** |
| --- | --- | --- | --- |
| *BCL11A-XL* | Fwd | CCAAACAGGAACACATAGCAGA | 52 |
|  | Rev | GAGCTCCATGTGCAGAACG |  |
| *GAPDH* | Fwd | AGCCACATCGCTCAGACAC | 60 |
|  | Rev | GCCCAATACGACCAAATCC |  |
| *GATA1* | Fwd | CACTGAGCTTGCCACATCC | 26 |
|  | Rev | ATGGAGCCTCTGGGGATTA |  |
| *HBA* | Fwd | GACCCGGTCAACTTCAAGC | 10 |
|  | Rev | AGAAGCCAGGAACTTGTCCA |  |
| *HBG* | Fwd | TGGATCCTGAGAACTTCAAGC | 72 |
|  | Rev | GCCACTGCAGTCACCATCT |  |
| *HBB* | Fwd | GCACGTGGATCCTGAGAACT | 61 |
|  | Rev | CACTGGTGGGGTGAATTCTT |  |
| *KLF1* | Fwd | ACACCAAGAGCTCCCACCT | 19 |
|  | Rev | GTAGTGGCGGGTCAGCTC |  |
| *RUNX1* | Fwd | CCTCAGAGTCAGATGCAGGA | 29 |
|  | Rev | GGAGAGGGATGGACAGAAGA |  |
| *SOX17* | Fwd | ACGCCGAGTTGAGCAAGA | 61 |
|  | Rev | TCTGCCTCCTCCACGAAG |  |

**Table S3. Primer sequences for Multiplex PCR analysis for hemoglobin E.**

| **Primer name** | **Sequence (5’🡪3’)** |
| --- | --- |
| HbE-Fc | TCC AAC TCC TAA GCC AGT GC |
| HbE-Rc | CCT GCC CAG GGC CTC |
| HbE-Rn | CGT GGA TGA AGT TGG TGG TA |
| HbE-Fm | CGA TCC TGA GAC TTC CAC ACT G |

**Table S4. Sequences for guide RNA (gRNA) cloning and validation.**

| **Primer name** | **Sequence (5’🡪3’)** | **PAM** |
| --- | --- | --- |
| gRNA1_Top | CACCGTG GAT GAA GTT GGT GGTA | AGG |
| gRNA1_Bottom | AAAC T ACC ACC AAC TTC ATC CAC |  |
| gRNA2_Top | CACCGGT GGTAAG GCC CTG GGC | AGG |
| gRNA2_Bottom | AAAC GCC CAG GGC CTT ACC ACC |  |
| gRNA3_top | CACC GCG TGG ATG AAG TTG GTG GTA | AGG |
| gRNA3_bottom | AAAC TAC CAC CAA CTT CAT CCA CGC |  |
| gRNA4_top | CACC GTG AAG TTG GTG GTA AGG CCC | TGG |
| gRNA4_bottom | AAAC GGG CCT TAC CAC CAA CTT CAC |  |
| gRNA5_top | CACC GAA GTT GGT GGT AAG GCC CT | GGG |
| gRNA5_bottom | AAAC AG GGC CTT ACC ACC AAC TTC |  |
| gRNA6_top | CACC GTTG GTG GTA AGG CCC TGG GC | AGG |
| gRNA6_bottom | AAAC GC CCA GGG CCT TAC CAC CAAC |  |
| gRNA7_top | CACC GTG GTA AGG CCC TGG GCA GGT | TGG |
| gRNA7_bottom | AAAC ACC TGC CCA GGG CCT TAC CAC |  |
| U6-Fwd | GAG GGC CTA TTT CCC ATG ATT CC |  |

**Table S5. Single-stranded DNA oligonucleotide (ssODN) template sequence for correcting HbE mutation**.

| ssODN (5’🡪3’) | CAAACAGACACCATGGTGCATCTGACTCCTGAGGAGAAGTCTGCCGTTACTGCCCTGTGGGGCAAGGTGAACGTGGATGAAGTTGGTGGTGAGGCCCTGGGCAGGTTGGTATCAAGGTTACAAGACAGGTTTAAGGAGACCAATAGAAACTGGGCATGTGGAGACAGAGAAGACTCTTGGG |
| --- | --- |

**Table S6. Primer sequences for off-target analysis**

| **OT** | **Forward primer** (**5’🡪3’)** | **Reverse primer (5’🡪3’)** | **Product size (bp)** |
| --- | --- | --- | --- |
| **1** | AACCAACCTGCTCACTGGAG | AGCCTTCACCTTAGGGTTGC | 309 |
| **2** | TGGAAGCAGGTGGACAGTTC | TGTGTAGGTTACCCAAGGCAC | 369 |
| **3** | TGCAGGTGTGTAGGTTGAGTC | CCAAACAACCCCTACCACCA | 352 |
| **4** | AACCGCATGGAGTCGTTCTT | ATGGCCCCGAACTAACAGTG | 307 |
| **5** | GAGTTGACCCGAAGAAGCTG | GAGTTGACCCGAAGAAGCTG | 412 |
