# Supplementary figures and images for: One-step genetic correction of hemoglobin E/beta-thalassemia patient-derived iPSCs by the CRISPR/Cas9 system
Source: Stem Cell Res Ther. 2018 Feb 26;9:46. doi: 10.1186/s13287-018-0779-3 (PMC5828150; doi:10.1186/s13287-018-0779-3)

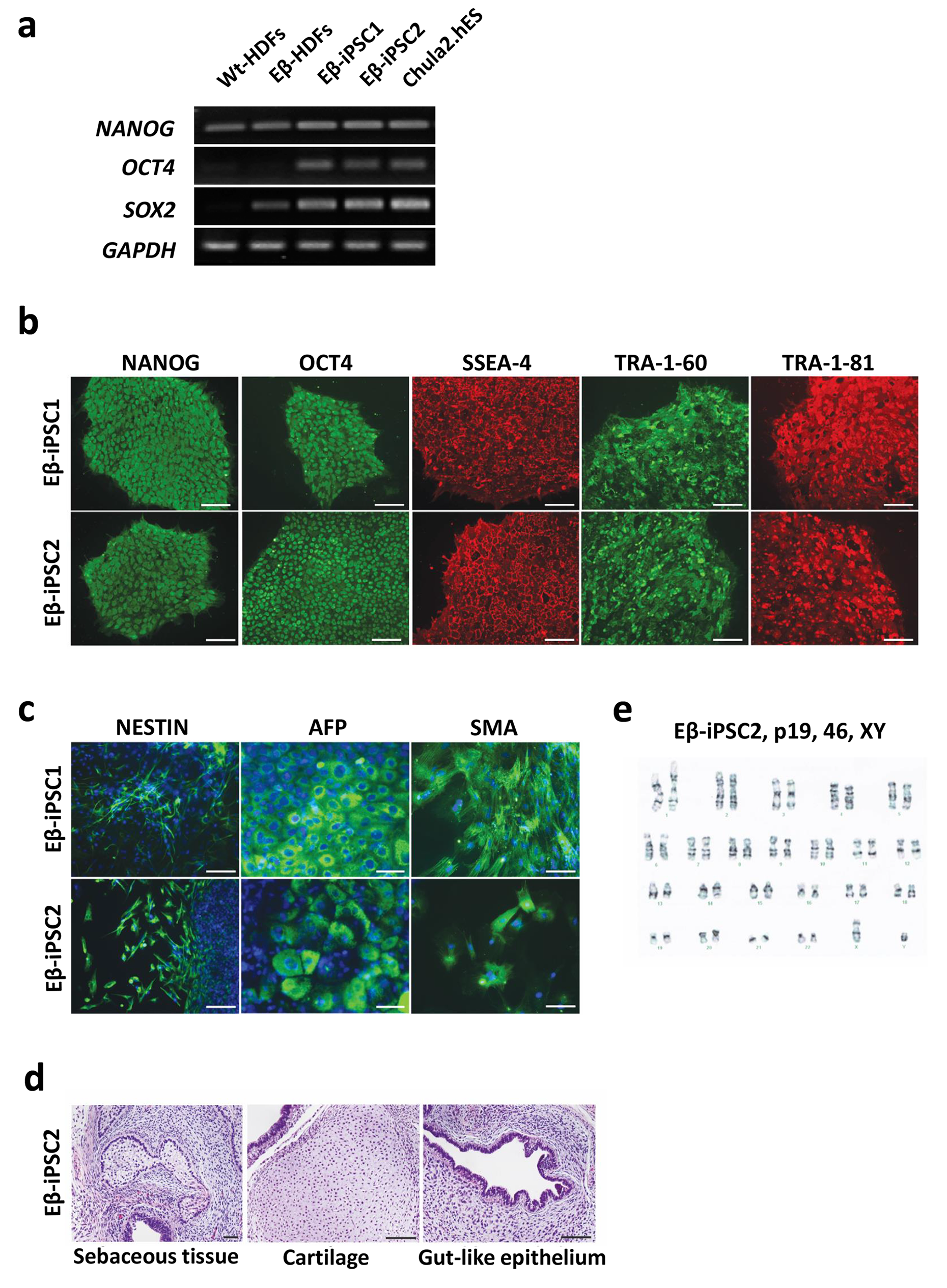

Supplement: Supplementary file 2 — Showing characterization of iPSCs derived from skin fibroblasts of a patient with hemoglobin E/beta-thalassemia. (a) Pluripotent gene expression of wild-type human dermal fibroblasts (wt-HDFs), parental human dermal fibroblasts (Eβ-HDFs) and Eβ-iPSCs compared with hESC line Chula2.hES, analyzed by RT-PCR. (b) Immunofluorescent staining shows expression of pluripotent markers NANOG, OCT4, SSEA-4, TRA-1-60 and TRA-1-81 in the Eβ-iPSC1 and Eβ-iPSC2 cells. Scale bars = 100 μm. (c) Immunofluorescent staining shows expression of lineage markers NESTIN (ectoderm), AFP (endoderm) and SMA (mesoderm) of differentiated embryoid bodies generated from the Eβ-iPSC1 and Eβ-iPSC2 cells. Scale bars: for NESTIN and SMA = 100 μm; for AFP = 50 μm. (d) Hematoxylin and eosin (H&E) staining of teratomas derived from the Eβ-iPSC2 cells at 8 weeks post implantation into nude mice. Teratomas contained tissues derived from three embryonic germ layers, sebaceous tissue (ectoderm), cartilage (mesoderm) and gut-like epithelium (endoderm). Scale bars = 100 μm. (e) Representative karyotypic analysis of the Eβ-iPSC2 cells at passage 19 shows normal karyotype (46, XY) (TIFF 9760 kb) [file 13287_2018_779_MOESM2_ESM.tif]

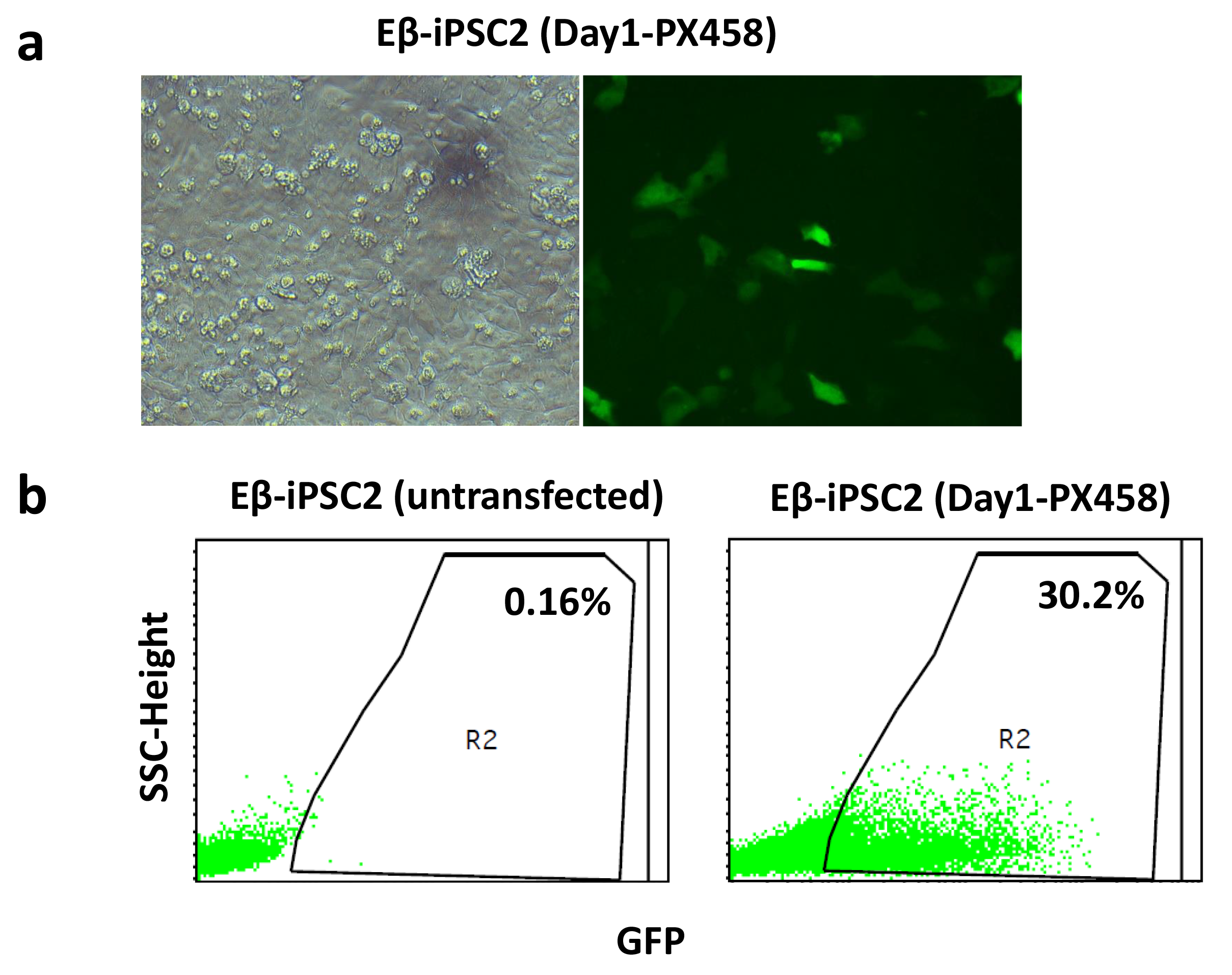

Supplement: Supplementary file 3 — Showing transfection efficiency of PX458 in the Eβ-iPSC2 cells. (a) Phase contrast and fluorescent images of the Eβ-iPSC2 cells 1 day post transfection with PX458. (b) Flow cytometry analysis of GFP-expressing cells in the untransfected cells (negative control) and the PX458 transfected cells (TIFF 4645 kb) [file 13287_2018_779_MOESM3_ESM.tif]

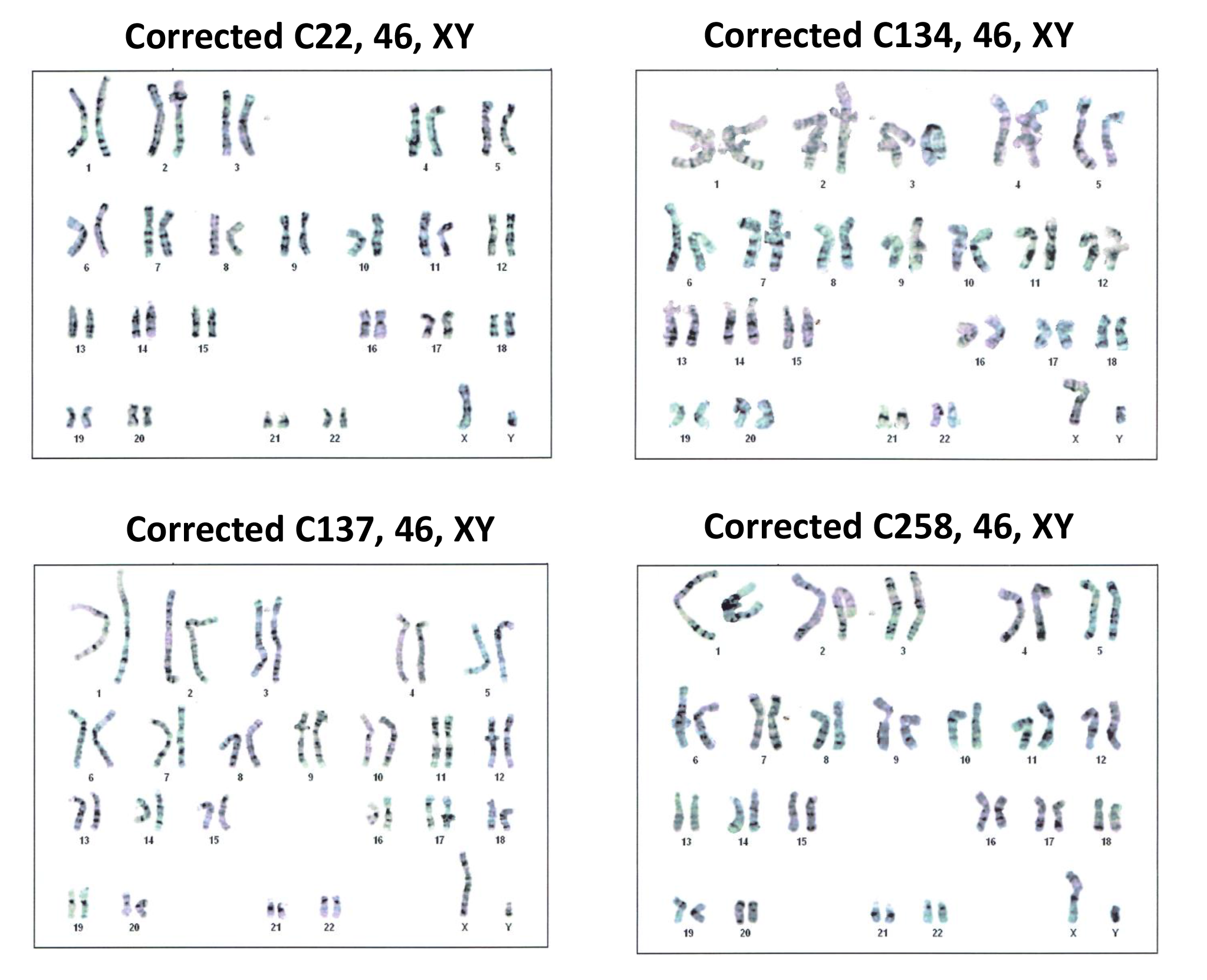

Supplement: Supplementary file 4 — Showing representative karyotypes of the corrected C22, C134, C137 and C258 cells, which exhibited normal karyotypes (46, XY) (TIFF 3596 kb) [file 13287_2018_779_MOESM4_ESM.tif]

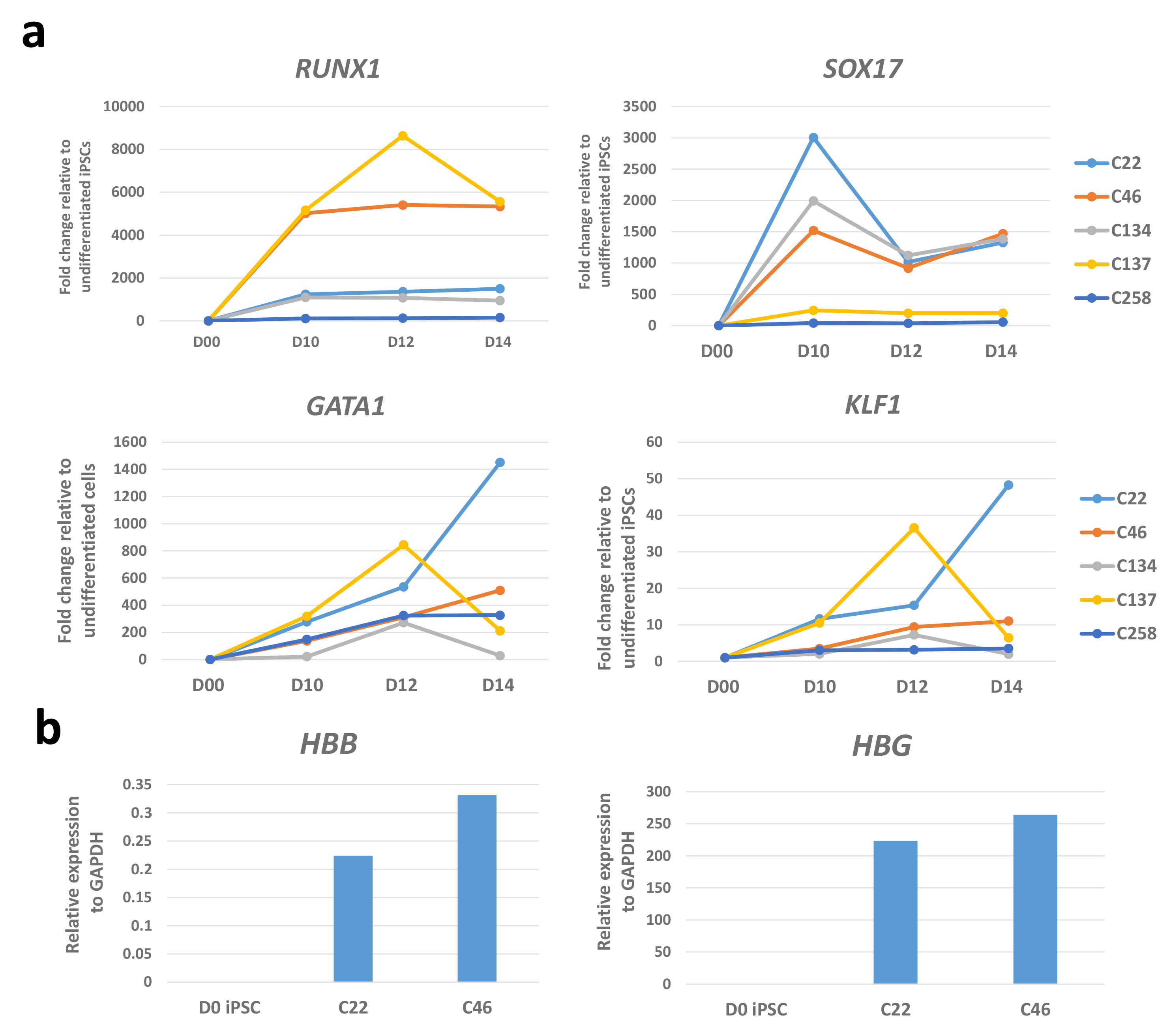

Supplement: Supplementary file 5 — Showing gene expression profile of the differentiated cells. (a) qRT-PCR analysis of hematopoietic and erythroid-specific markers: RUNX1, SOX17, GATA1 and KLF1. Data presented as relative expression to day 0 of each sample, N = 2. (b) qRT-PCR analysis of fetal (HBG) and adult (HBB) globin gene expressions of day 0 normal iPSCs (D0 iPSC) and BFU-E and CFU-E colonies of the corrected C22 and C46 cells on day 14 of culture in MethoCult media. Data presented as relative expression to GAPDH, N = 2 (TIFF 1576 kb) [file 13287_2018_779_MOESM5_ESM.tif]
